# Improved Ionization Potential Depression Model Incorporating Dynamical Structure Factors and Electron Degeneracy for Non-Ideal Plasma Composition

**DOI:** 10.3390/e27030253

**Published:** 2025-02-27

**Authors:** Yeldos Seitkozhanov, Karlygash Dzhumagulova, Erik Shalenov

**Affiliations:** 1 Department of General Physics, Satbayev University, Almaty 050013, Kazakhstan; shalenov.erik@mail.ru; 2 Department of Plasma Physics, Nanotechnology and Computer Physics, Al-Farabi Kazakh National University, Almaty 050040, Kazakhstan; 3 Institute of Experimental and Theoretical Physics, Al-Farabi Kazakh National University, Almaty 050040, Kazakhstan

**Keywords:** non-ideal plasma, strongly coupled plasma, plasma composition, ionization potential depression, dynamical structure factor, Saha equation, high-energy-density physics, Mott transition

## Abstract

In this work, we present an improved model for ionization potential depression (IPD) in dense plasmas that builds upon the approach introduced by Lin et al., which utilizes a dynamical structure factor (SF) to account for ionic microfield fluctuations. The main refinements include the following: (1) replacing the Wigner–Seitz radius with an ion-sphere radius, thereby treating individual ionization events as dynamically independent; (2) incorporating electron degeneracy through a tailored interpolation between Debye–Hückel and Thomas–Fermi screening lengths. Additionally, we solve the Saha equation iteratively, ensuring self-consistent determination of the ionization balance and IPD corrections. These modifications yield significantly improved agreement with recent high-density and high-temperature experimental data on warm dense aluminum, especially in regimes where strong coupling and partial degeneracy are crucial. The model remains robust over a broad parameter space, spanning temperatures from 1 eV up to 1 keV and pressures beyond the Mbar range, thus making it suitable for applications in high-energy-density physics, inertial confinement fusion, and astrophysical plasma research. Our findings underscore the importance of accurately capturing ion microfield fluctuations and electron quantum effects to properly describe ionization processes in extreme environments.

## 1. Introduction

The investigation of dense plasmas is critical to various scientific fields, including astrophysics, fusion energy research, materials science, high-energy density physics, planetary science, and nuclear physics. This exploration enhances our understanding of complex phenomena such as stellar evolution, energy production mechanisms, the behavior of materials under extreme conditions, planetary interiors, and nuclear reaction processes. Recent advancements in experimental techniques have significantly strengthened the ability to validate and refine theoretical models. These developments have amplified interest in thoroughly examining the thermodynamic, optical, and transport properties of dense plasmas to bridge the gap between experimental observations and theoretical predictions [1,2,3,4,5,6,7,8]. A key aspect of these investigations involves accurately modeling the ionization equilibrium within such plasmas, as this plays a crucial role in describing their behavior. There are numerous approaches for determining ionization equilibrium, broadly categorized into “physical” and “chemical” pictures. The physical picture is theoretically rigorous as it incorporates many-body Coulomb interactions among electrons and nuclei. However, this approach becomes mathematically and computationally challenging as plasma density rises, resulting in significant complexity in practical applications [9]. Methods such as finite-temperature density functional theory [10,11], classical molecular dynamics [12,13], and Monte Carlo simulations [14,15] are commonly used within this framework but are computationally intensive and can face limitations in highly coupled regimes.

Conversely, the chemical picture conceptualizes the ionization equilibrium in terms of chemical reactions, simplifying the mathematical treatment while retaining physical insight. This approach utilizes the Saha equation [16], which quantitatively links the degree of ionization to plasma temperature, pressure, and ionization energies of constituent particles. While highly effective for ideal plasmas, the Saha equation must be adapted for application to non-ideal, dense plasma conditions. In dense plasmas, non-ideal effects—primarily due to strong interparticle interactions—require corrections to the ionization energy. One significant effect is the reduction in ionization energy due to induced screening, known as ionization potential depression (IPD). This phenomenon has drawn considerable attention, particularly with recent high-energy laser-based experimental investigations such as those at the X-ray Free Electron Laser Linac Coherent Light Source (XFEL LCLS) [1,2] and laser-driven compression platforms like Orion (UK) [4] and the National Ignition Facility (NIF) [3,6,7]. These experiments have highlighted discrepancies in the performance of established IPD models, including Stewart–Pyatt (SP) [17] and Ecker–Kroll (EK) [18]. Specifically, XFEL results suggest that the SP model underestimates the IPD, while the modified EK model provides a closer match to experimental data. Conversely, some laser-driven compression studies indicate that only the SP model successfully describes the behavior of hot, dense aluminum plasmas [4]. For a comprehensive examination of these interpretations and the applicability of various models, refer to discussions in [19,20,21]. A range of theoretical approaches has emerged to capture the IPD across different coupling regimes, incorporating advanced quantum statistical methods and simulation techniques [8,19,20,21,22,23,24,25]. In this work, we apply a recently developed model by Lin et al., which uses the dynamical structure factor (SF) to account for IPD [20,26]. This approach, grounded in quantum statistical mechanics, has demonstrated improved accuracy for IPD predictions. Lin et al. initially employed the SF as a correction for ionic microfield fluctuations within average atom models. However, in our study, we substitute the Wigner–Seitz radius commonly used in average-atom frameworks with the ion sphere radius. This modification aligns with the view that individual ionization processes are dynamically independent, not directly affecting the overall ionization equilibrium of other ions or the plasma itself [21].

Additionally, we incorporate electron degeneracy through an interpolation formula and adapt the algorithm for solving the Saha equation [27,28] to align with our current IPD model. This comprehensive approach allows for a more precise description of ionization equilibrium in non-ideal plasmas, enhancing our understanding of dense plasma behavior and contributing to a consistent theoretical framework that aligns with experimental observations.

The present work is organized as follows: In Section 2, we describe the theoretical framework underlying the calculation of plasma composition, including the implementation of the Saha equation with non-ideal corrections. Section 2.1 details the approach to solving the ionization balance using the modified Saha equation. Section 2.2 introduces modifications to the ionization potential depression model, incorporating dynamical structure factors as corrections to average-atom models. The interpolation formula used for screening lengths, bridging the gap between classical Debye and quantum Thomas–Fermi regimes, is discussed in Section 2.3

Section 3 focuses on the results, examining the IPD in Section 3.1 and plasma composition in Section 3.1 across various temperature and density conditions. We provide a detailed comparison with existing models and experimental data, highlighting the advantages of our method. Specific comparisons with the works of Perrot and Dharma-Wardana [12], and Kim [29], are presented to contextualize our findings within the broader field.

Finally, Section 4 summarizes the key findings of this study, outlining the implications of the results for plasma modeling and suggesting potential directions for future research.

## 2. Methods

### 2.1. Saha Equation

Accurate determination of plasma composition requires self-consistent solutions of the Saha equation under conditions where non-ideal and quantum effects become significant. The standard Saha equation [16] for ionization equilibrium can be written as follows [16]:

(1) $$\frac{n_{i+1}n_{e}}{n_{i}}=\frac{2U_{i+1}}{U_{i}}{\left(\frac{2\pi m_{e}k_{B}T}{h^{2}}\right)}^{3/2}\exp\left(-\frac{I_{i}^{\mathrm{eff}}}{k_{B}T}\right),$$

where $n_{i}$ and $U_{i}$ are the number density and temperature-dependent partition function of the *i*-fold ionized state (with i=0 representing neutral atoms), respectively. In this work, the partition functions have been approximated to be the statistical weights of the ground states. $n_{e}$ is the number density of free electrons. The effective ionization energy is given by $I_{i}^{\mathrm{eff}}=I_{i}-\Delta I_{i}$, where $I_{i}$ is the ionization energy for the *i*-fold ionized state and $\Delta I_{i}$ is the ionization potential depression. Here, $m_{e}$ is the electron mass, $k_{B}$ is the Boltzmann constant, and *T* is the temperature. To find a self-consistent solution for the plasma’s ionization state, the Saha equation is used in conjunction with the following conditions:

Electroneutrality condition:

(2) $$\sum_{i=1}^{Z_{\max}}in_{i}=n_{e},$$

Conservation of nuclei:

(3) $$\sum_{i=0}^{Z_{\max}}n_{i}=n_{H}=\mathrm{const},$$

where $Z_{\max}$ is the highest ionization stage achievable under the plasma conditions and $n_{H}$ is the number density of heavy particles (nuclei). These equations, together with the Saha equation, form a system of nonlinear algebraic equations. The solution to this system is obtained using the algorithm proposed by Zaghloul et al. [27]. Replacing number densities with the relative fractions ($\alpha_{i}=\frac{n_{i}}{n_{H}}$) of ions with charge *i* and ionization degree ($\alpha_{e}=\frac{n_{e}}{n_{H}}$), Equations (1)–(3) can be reformulated as follows:

(4) $$\frac{\alpha_{i+1}\alpha_{e}n_{H}}{\alpha_{i}}=2\frac{U_{i+1}}{U_{i}}{\left(\frac{2\pi m_{e}k_{B}T}{h^{2}}\right)}^{3/2}\exp\left(-\frac{I_{i}}{k_{B}T}\right)=f_{i+1},\;i=0,1,\ldots ,\left(Z_{\max}-1\right),$$

(5) $$\sum_{i=0}^{Z_{\max}}\alpha_{i}=1,$$

(6) $$\sum_{i=1}^{Z_{\max}}i\alpha_{i}=\alpha_{e}.$$

The recurrence relation comes out of Equation (4):

(7) $$\alpha_{i+1}=\frac{\alpha_{i}}{\alpha_{e}n_{H}}f_{i+1}.$$

Substituting (7) into (6), we can obtain the following expression for $\alpha_{0}$:

(8) $$\alpha_{0}=\alpha_{e}{\left(\sum_{i=1}^{Z_{\max}}\frac{i\prod_{j=1}^{i}f_{j}}{{\left(\alpha_{e}n_{H}\right)}^{i}}\right)}^{-1}.$$

Substituting this relation with the recurrence relation (7) into Equation (5), one can derive a transcendental equation for $\alpha_{e}$:

(9) $$1-\alpha_{e}{\left(\sum_{i=1}^{Z_{\max}}\frac{i\prod_{j=1}^{i}f_{j}}{{\left(\alpha_{e}n_{H}\right)}^{i}}\right)}^{-1}\left(1+\sum_{i=1}^{Z_{\max}}\frac{\prod_{j=1}^{i}f_{j}}{{\left(\alpha_{e}n_{H}\right)}^{i}}\right)=0.$$

Solving this transcendental equation for $\alpha_{e}$ allows us to determine the plasma composition by substituting back into Equations (7) and (8) to find each $\alpha_{i}$.

### 2.2. IPD Models and Dynamical Structure Factor Approach

At certain temperatures and densities, particle interactions and quantum mechanical effects significantly influence plasma behavior. These effects modify the ionization equilibrium by shifting the continuum boundary, effectively reducing the ionization energy of particles. In the Saha equation, these corrections for plasma non-ideality are represented as ionization potential depression.

Several models exist for calculating IPD. For low-density and high-temperature plasmas, the approach introduced by Griem [30] is commonly employed, where IPD is expressed as

(10) $$\Delta I_{i}=\frac{\left(i+1\right)e^{2}}{4\pi \varepsilon_{0}\lambda_{D}},$$

where $\lambda_{D}=\sqrt{\frac{\varepsilon_{0}k_{B}T}{e^{2}\left(n_{e}+\sum_{i}^{Z_{\max}}i^{2}n_{i}\right)}}$ is the Debye screening length. Here, *e* is the electron charge and $\varepsilon_{0}$ is the vacuum permittivity. This formulation is often termed the Debye–Hückel (DH) model.

For dense plasmas, models based on the average-atom approximation, such as the ion sphere (IS), Stewart–Pyatt (SP), and Ecker–Kroll (EK) models, are widely used. The IS model considers ions as point charges surrounded by an electron cloud within a spherical region, with the IPD given by

(11) $$\Delta I^{\mathrm{IS}}=C_{\mathrm{IS}}\frac{z^{*}e^{2}}{4\pi \varepsilon_{0}r_{\mathrm{WS}}},$$

where $r_{\mathrm{WS}}={\left(\frac{3}{4\pi n_{i}}\right)}^{1/3}$ is the Wigner–Seitz radius and $z^{*}$ is an average ion charge. The constant $C_{\mathrm{IS}}=\frac{9}{5}$ is taken as determined in [31].

The SP model interpolates between DH and IS limits [17] and is implemented in plasma simulation codes such as CRETIN [32], FLYCHK [33], and LASNEX-DCA [34]. The IPD in this model is

(12) $$\Delta I_{i}^{\mathrm{SP}}=\frac{3}{2}\frac{\left(i+1\right)e^{2}}{4\pi \varepsilon_{0}r_{i}^{\mathrm{IS}}}\left[{\left(1+s^{3}\right)}^{2/3}-s^{2}\right]$$

where $r_{i}^{\mathrm{IS}}={\left(\frac{3i}{4\pi n_{e}}\right)}^{1/3}$ is the ion sphere radius and $s={\left(\kappa_{D}r_{i}^{\mathrm{IS}}\right)}^{-1}$, where $\kappa_{D}$ is the inverse Debye screening length. The SP model, while often categorized under average atom models due to its overall treatment of plasma properties, interpolates between the collective behavior of the plasma and specific interactions involving individual ions. This approach allows for a more nuanced IPD calculation that reflects both the averaged environment and individual ionic characteristics.

The EK [18] model divides the plasma system into two regions based on the total number density of particles in plasma $n=n_{e}+n_{i}$. In the low-density region ($n<n_{\mathrm{cr}}$), the DH model is used (10). In the high-density region ($n\geq n_{\mathrm{cr}}$), the IPD takes the form

(13) $$\Delta I_{i}^{\mathrm{EK}}=C_{\mathrm{EK}}\frac{\left(i+1\right)e^{2}}{4\pi \varepsilon_{0}r_{\mathrm{EK}}},$$

where $r_{\mathrm{EK}}={\left(\frac{3}{4\pi n}\right)}^{1/3}$ is the average interparticle distance and $C_{\mathrm{EK}}$ is an adjustable parameter, highlighting the model’s empirical nature. $n_{\mathrm{cr}}$ is the critical number density, given by

(14) $$n_{\mathrm{cr}}=\frac{3}{4\pi }{\left(\frac{k_{B}T_{e}}{Z^{2}e^{2}}\right)}^{3}.$$

Debate over the applicability of these models in interpreting experimental data [1,2,3,7] has led to the development of new IPD calculation methods [19,20,21,23,35]. In this paper, we used the formalism proposed by Lin et al. [20,26], which refines mean-field approaches by incorporating ion microfield fluctuations through a dynamical structure factor (SF). In contrast to Lin et al. [26], who developed their model for multicomponent plasmas—with the ion-sphere radius used to characterize impurity ions embedded in a charged environment and electron degeneracy accounted for directly via the RPA dielectric function—our approach is specifically designed for a one-component plasma. Here, each ion is assigned an individual ion-sphere radius $r_{i}^{\mathrm{IS}}$ to more accurately capture the local ionic environment. Furthermore, while Lin et al. incorporate electron degeneracy effects directly into the screening function $q_{sc}\left(k\right)$ via the RPA dielectric function, our model introduces these effects through an interpolation for the inverse screening length. This modification reduces computational complexity while still preserving the key physics of quantum degeneracy. This refined approach is further elaborated in the formulation of the ionic dynamical structure factor below.

The ionic part of the dynamical structure factor can be expressed in terms of a static one based on the use of the plasmon pole approximation, which is justified by the significantly slower motion of ions compared to electrons. The IPD calculated using this approach is given by

(15) $$\Delta I_{i}=\frac{\left(i+1\right)e^{2}\kappa_{\mathrm{eff}}^{2}}{2\pi^{2}\varepsilon_{0}r_{\mathrm{WS}}}\int_{0}^{\infty }\frac{dk_{0}}{k_{0}^{2}}S_{ii}^{ZZ}\left(k_{0}\right),$$

where $k_{0}=\frac{k}{k_{F,i}}$ is the reduced wavenumber with $k_{F,i}={\left(3\pi^{2}n_{i}\right)}^{1/3}$ being the Fermi wavenumber of the ions. $S_{ii}^{ZZ}\left(k_{0}\right)$ is the reduced ionic charge–charge static structure factor. In this work, we improve the approach of Lin by incorporating the radius of individual ions and considering the degeneracy of electrons in the calculation of IPD. This approach is inspired by Crowley’s work [21], which asserts that ionization processes are dynamically independent, and each process does not directly affect the overall ionization equilibrium of the plasma. As described by Crowley, individual ionization events are treated as quasi-static transitions that occur independently, without altering the equilibrium state of other ions or the plasma as a whole.

To align with Crowley, we replaced the Wigner–Seitz radius $r_{\mathrm{WS}}$, which characterizes the plasma as a whole, with the ion-sphere radius $r_{i}^{\mathrm{IS}}$, to better represent the local environment surrounding each ion [21]. The relationship between these two radii is given by

(16) $$r_{i}^{\mathrm{IS}}={\left(\frac{i}{\alpha_{e}}\right)}^{1/3}r_{\mathrm{WS}},$$

The effective inverse screening length in Equation (15) is taken in an approximate form as

(17) $$\kappa_{\mathrm{eff}}^{2}={\frac{r_{\mathrm{WS}}{\tilde{\kappa }}_{i}}{k_{F,i}}}^{2}={\left(\frac{4}{9\pi }\right)}^{1/3}r_{\mathrm{WS}}^{2}{\tilde{\kappa }}_{i}^{2}=\frac{3\Gamma_{i}}{\sqrt{{\left(\frac{9\pi }{4}\right)}^{2/3}+3\Gamma_{i}}},$$

where $\Gamma_{i}=\frac{i^{2}e^{2}}{4\pi \varepsilon_{0}r_{i}^{\mathrm{IS}}}$ is the ionic coupling parameter. This expression reproduces the DH limit for weakly coupled plasmas and aligns with numerical solutions of the normalization relation provided below.

The normalization relation that determines the effective screening length ${\tilde{\kappa }}_{i}$ for strongly coupled systems is given by

(18) $$\int_{0}^{\infty }dx\;x^{2}\left[1-\exp\left(-\frac{\Gamma_{i}}{x}\exp\left(-{\tilde{\kappa }}_{i}\Gamma_{i}r_{i}^{\mathrm{IS}}x\right)\right)\right]=\frac{1}{3},$$

The ionic charge–charge structure factor, which accounts for the screening cloud of slowly moving electrons that follow the ionic motion, is defined as

(19) $$S_{ii}^{ZZ}\left(k\right)={\left(1-q_{\mathrm{sc}}\left(k\right)\right)}^{2}S_{ii}\left(k\right),$$

where $q_{\mathrm{sc}}\left(k\right)$ is the screening function, which includes correlations between bound and free electrons. The ion–ion static structure factor $S_{ii}\left(k\right)$ and $q_{\mathrm{sc}}\left(k\right)$ are obtained using the analytical model described in Ref. [36].

### 2.3. Inclusion of the Electron Degeneracy

In dense plasmas, the behavior of $q_{\mathrm{sc}}\left(k\right)$ is influenced by electron degeneracy, a phenomenon arising from the quantum mechanical properties of electrons at high densities. The classical Debye–Hückel (DH) theory is insufficient to account for these effects, and the Thomas–Fermi (TF) screening length must be introduced instead [37]. The screening function $q_{\mathrm{sc}}\left(k\right)$ presented in Ref. [36] is based on the Debye inverse screening length. In this work, $q_{\mathrm{sc}}\left(k\right)$ is extended to incorporate the inverse screening length $k_{Y}$, which interpolates between the DH and TF regimes, as described in Ref. [38]:

(20) $$k_{Y}={\left[\frac{1}{2}k_{TF}^{2}\theta^{1/2}I_{-1/2}\left(\eta \right)\right]}^{1/2}$$

where $I_{n}\left(\eta \right)$ is the Fermi integral of order *n*, defined as

(21) $$I_{n}\left(\eta \right)=\int_{0}^{\infty }\frac{t^{n}}{e^{\left(t-\eta \right)}+1}\;dt,$$

with $\eta =\frac{\mu_{e}}{k_{B}T_{e}}$, where $\mu_{e}$ is the electron chemical potential. The TF inverse screening length is given by

(22) $$k_{TF}=\frac{\sqrt{3}\omega_{p}}{v_{F}}$$

where $\omega_{p}=\sqrt{\frac{n_{e}e^{2}}{m_{e}\varepsilon_{0}}}$ is the electron plasma frequency, $v_{F}=\frac{\hbar k_{F}}{m_{e}}$ is the Fermi velocity, $k_{F}={\left(3\pi^{2}n_{e}\right)}^{1/3}$ is the Fermi wavevector, $\theta =\frac{k_{B}T_{e}}{E_{F}}$ is the degeneracy parameter, and $E_{F}=\frac{\hbar^{2}k_{F}^{2}}{2m_{e}}$ is the electron Fermi energy.

The chemical potential $\mu_{e}$ is determined by inverting the Fermi integral. In [39], an interpolation formula is proposed to simplify this process, eliminating the need to evaluate the Fermi integral explicitly:

(23) $$\frac{\mu_{e}\left(n_{e},T\right)}{k_{B}T}=\left\{\begin{array}{cc} \ln y_{e}+0.3536y_{e}-0.00495y_{e}^{2}+0.000125y_{e}^{3}, & y_{e}<5.5,\\ 1.209y_{e}^{2/3}-0.6803y_{e}^{-2/3}-0.85y_{e}^{-2}, & y_{e}\geq 5.5, \end{array}\right.$$

where $y_{e}=\frac{n_{e}\Lambda_{e}^{3}}{2s_{e}+1}$, $\Lambda_{e}={\left(\frac{2\pi \hbar^{2}}{m_{e}k_{B}T}\right)}^{1/2}$ is the de Broglie thermal wavelength, and $s_{e}$ is the spin of the electron.

Thus, the model presented in this paper is based on the following equations and approximations. To determine the composition of the dense quantum plasma, the Saha equation system (4) with additional conditions (5) and (6) is solved. Equation (15) was chosen as the expression for IPD, which made it possible to take into account the fluctuations of the ion microfield. However, in Equation (15), the Wigner–Seitz radius in our model is replaced by the ionospheric radius (16). The structure factor included in (15) is determined based on the method described in Ref. [36]. In this work, the inverse screening length, necessary for calculating the structure factor, takes into account the effects of both screening and degeneracy (see Equation (20)).

### 2.4. The Algorithm for Solving the Saha Equation

To solve the Saha equation after including the ionization potential depression (IPD), the next iterative procedure is applied:

1. **Initial guess:** Start with an initial guess for $\alpha_{e}$. Also, find $\Delta I_{i}$ by using another model explicitly expressed in terms of $n_{H}$ and $\alpha_{e}$. For instance,
  $$\Delta I_{i}=6.96\times 10^{-7}\;\cdot \;\sqrt[{3}]{n_{e}\;\left[{\mathrm{cm}}^{-3}\right]}\;Z_{\mathrm{eff}}^{2/3}\;\left[\mathrm{eV}\right]$$
  where $Z_{\mathrm{eff}}=\left(i+1\right)$ is the effective charge state of the ion [40].
2. **Calculate $\alpha_{e}$ and $\alpha_{i}$**: Calculate $\alpha_{e}$ from Equation (9) with $I_{i}^{eff}$=$I_{i}-\Delta I_{i}$. Calculate $\alpha_{i}$ using the recurrence relation (7).
3. **Recompute IPD:** Use the $\alpha_{e}$ and $\alpha_{i}$ in Equations (15) and (16) according to our model to obtain new values for $\Delta I_{i}$.
4. **Check convergence:** Compare the recalculated $\Delta I_{i}$ with the previous value. If the difference exceeds the desired accuracy, use the recalculated $\Delta I_{i}$ as the guess values.
5. **Converge:** Iterate until the difference between the calculated and guess $\Delta I_{i}$ falls below the predefined accuracy.

## 3. Results and Discussion

### 3.1. Ionization Potential Depression

In this subsection, we present the IPD, calculated within the frameworks of the different models. The model that we present in this work (SF-IF) is described in the previous section.

Our model makes it possible to obtain adequate results in a wide range of temperatures and concentrations, since the interpolated transition from the DH screening length to the Thomas–Fermi screening length makes it possible to correctly take into account the degeneracy with increasing density and decreasing plasma temperature. In this regard, to demonstrate the adequacy of our approach, we present here a comparison of our results with the available data obtained on the basis of other models.

**Figure 1:**
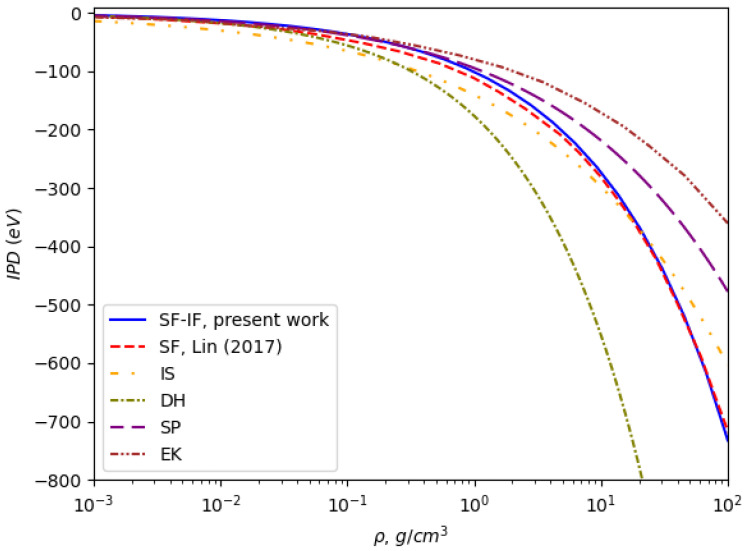
Ionization potential depression for Al^11+^ at a temperature of 600 eV as a function of plasma density, comparing the predictions of various models. The results highlight the differences in IPD magnitudes between Debye–Hückel [21], Ecker–Kroll [18], Stewart–Pyatt [17], ion sphere (IS) [31], Lin’s dynamical structure factor (SF) [20], and the present work (SF-IF) approaches.

In Figure 1, one can see the ionization potential depressions for the Al^11+^ ion as the function of the aluminum plasma density at a plasma temperature of 600 eV for six models. The Debye–Hückel model shows the largest magnitudes for IPD for non-ideal plasma. On the contrary, the Ecker–Kroll model gives the smallest ones. Our calculations lie lower than Lin’s calculations (SF) up to densities of about 20 g/cm^3^. At low and medium densities, they coincide with the Stewart–Pyatt data.

**Figure 2:**
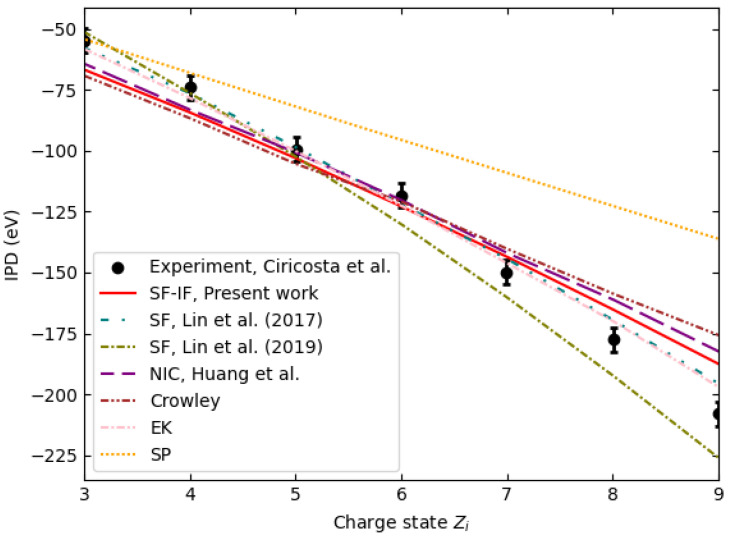
Ionization potential depression at T=100eV and a solid density of $2.7\;g\;cm^{-3}$ as a function of charge state. The comparison includes experimental data [2] and theoretical predictions from the SP [17], modified EK [18], Crowley [21], Huang et al. [42], and SF models [20,26] alongside the results of the present work (SF-IF).

Several experiments serve as a framework to verify the correctness of IPD calculations. Figure 2 illustrates the discrepancies between theoretical models and experimental observations [2] of IPD at a solid density of 2.7 $g\;cm^{-3}$. The charge state and electron distribution in the plasma created by the femtosecond laser are not in equilibrium in this experiment. However, the quasi-stationary condition, where the time scale of evolution of characteristic changes in plasma parameters is large enough to ensure that the ion system can follow and achieve approximately stationary conditions during the system’s time evolution, can be fulfilled. According to [41], for the quasi-stationarity condition to be fulfilled, the confinement parameter for the K-shell should be $n_{e}\tau_{\mathrm{plasma}}\approx 3\;\cdot \;10^{11}\;\;cm^{-3}s$. $\tau_{\mathrm{plasma}}$ is the plasma confinement parameter, which, in this case, can be considered as the duration of the laser pulse. For the experiment in question, the pulse duration and electron density are approximately 80 fs and $13.8\;\cdot \;10^{22}\;\;cm^{-3}$, respectively, resulting in a confinement parameter of $1.1\;\cdot \;10^{11}\;\;cm^{-3}s$. This ensures that the quasi-stationary state is achieved.

Based on this quasi-stationary condition, it can be assumed that collisional processes dominate over time, allowing the plasma subsystems to locally equilibrate. This supports the validity of assuming local thermodynamic equilibrium (LTE) for describing ionization potential depression in dense plasmas. The experiments by Ciricosta et al. [1] provide further evidence for this assumption. In these experiments, the stabilization of charge states and electron distributions over time indicates that LTE conditions are approximately achieved. This also aligns with the framework proposed by Lin et al. [20]

Despite these experimental conditions, traditional models, such as the Stewart–Pyatt [17] and Ecker–Kroll [18] formulations, fail to consistently match experimental data. For example, the SP model underestimates the IPD for highly charged ions in strongly coupled regimes, while the modified EK (mEK) model provides a closer fit to specific experimental observations. However, as noted by Rosmej et al. [23], the mEK model, with its adjustment of the parameter $C_{\mathrm{EK}}$ in Equation (13), serves more as a particular fit to specific datasets rather than a generalizable model. This limitation highlights the challenges in describing IPD across diverse plasma conditions using traditional frameworks.

The work of Huang et al. [42] introduces a self-consistent approach that accounts for non-ideal characteristics (NIC) in the partition functions of free electrons, thereby extending the ionization balance equation to dense regimes. This framework successfully interpreted opacity measurements of dense $C_{9}H_{10}$ plasma and has now been applied to IPD predictions for aluminum and gold plasmas, with their predictions aligning closely with our SF-IF model—particularly for the lower charge states.

Moreover, their investigation reveals that under XFEL irradiation, the free electrons do not fully thermalize because the high charge states are generated later in the laser pulse, when the free electron distribution is still evolving. To capture this non-equilibrium behavior, they introduce an “effective” electron temperature for the higher charge states ($Al^{7+}$–$Al^{9+}$), which better represents the lower energy of the free electrons surrounding these ions. Remarkably, theoretical predictions using this effective temperature yield IPD values that closely match the experimental data (see dashed line in Figure 2). This combined approach underscores the importance of incorporating both non-ideal characteristics and non-equilibrium effects to accurately predict IPD in dense plasmas.

Crowley [21] emphasizes the importance of distinguishing between equilibrium IPD, which reflects thermodynamic free energy differences, and non-equilibrium IPD, observed under conditions where the plasma environment cannot dynamically respond to ionization events. While such a distinction is critical for interpreting IPD in non-LTE scenarios, the present work assumes local thermodynamic equilibrium (LTE), where ionization processes are governed by collective plasma behavior and equilibrium thermodynamics. Under these conditions, discrepancies in traditional models like Stewart–Pyatt and modified Ecker–Kroll (mEK) arise from their inability to incorporate critical factors, such as ion microfield fluctuations, electron degeneracy, and dynamic plasma structure. By addressing these limitations, our approach provides a more physically consistent description of IPD in LTE plasmas compared to Crowley’s model, which significantly underestimates IPD for high charge states.

### 3.2. Plasma Composition

**Figure 3:**
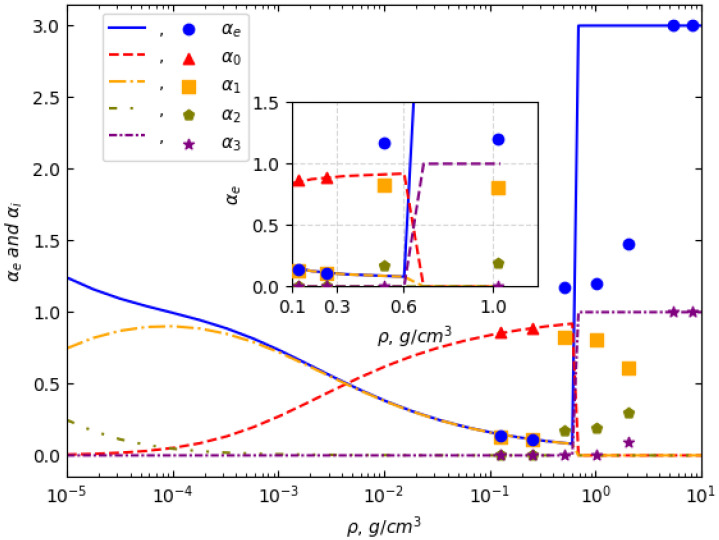
The composition of aluminum plasma at T=1.5eV as a function of plasma density. The comparison includes results from the present work and the calculations of Perrot and Dharma-Wardana [12].

Implementing the IPD calculations based on our SF-IF model, we have obtained the composition of aluminum plasma in a wide range of temperatures and concentrations. The results of calculating the degree of ionization and the reduced ion concentrations at a plasma temperature of 1.5 eV are shown in Figure 3 as lines. At this temperature of 1.5 eV, there are data from Perrot and Dharma-Wardana [12], who performed a computer experiment using the Density Functional Theory (DFT) calculations. Their data are presented using circles, triangles, etc.

As seen from this figure, the Mott transition point (the density value at which pressure ionization begins) in our calculations coincides with the Mott transition point in the computer experiment. Perrot and Dharma-Wardana’s work shows that the degree of ionization before the onset of pressure ionization decreases to small values, approximately $\alpha_{e}=0.1$, which is indicated as unstable. The values of the degree of ionization and the reduced concentrations calculated in our work before the Mott transition also practically coincide with the data of Perrot and Dharma-Wardana. The transition itself in the computer experiment is not as sharp as in theoretical calculations. However, the experimental data on determining the electrical conductivity during pressure ionization indicate a sharp increase in the degree of ionization [43].

**Figure 4:**
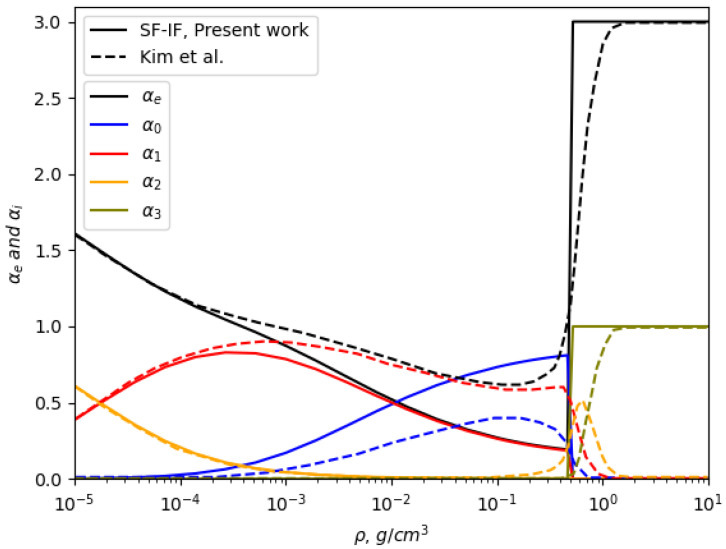
The composition of the aluminum plasma at T=1.73eV(20,000K) as a function of plasma density. Dashed lines represent the results of Kim and Kim [29].

Figure 4 illustrates the composition of aluminum plasma at a temperature of 1.73 eV (20,000K) as a function of plasma density, comparing our results with those obtained by Kim and Kim [29]. At lower densities, where the Debye–Hückel limit dominates, both models exhibit a gradual decrease in $\alpha_{e}$, reflecting the weakly coupled nature of the plasma. This agreement at low densities suggests that both approaches adequately capture the plasma’s ionization behavior under conditions where ionic interactions and microfield effects are less pronounced.

Notable discrepancies, however, arise at higher densities. In our model, the phase transition occurs at a lower $\alpha_{e}$, showing suppressed ionization states initially and a sharp transition in $\alpha_{e}$ from approximately 0.2 to 3 as the density increases. In contrast, Kim and Kim’s results demonstrate a smoother progression, with $\alpha_{e}$ starting at 0.8 and gradually increasing without the abrupt transition observed in our work.

Kim and Kim’s methodology relies heavily on semi-empirical corrections to IPD, employing excess free energy models derived from Monte Carlo and hypernetted-chain calculations. While these corrections are computationally efficient and account for general plasma interactions, they do not explicitly capture the microfield fluctuations and local screening effects that dominate in dense plasmas. The reliance on semi-empirical adjustments leads to a less pronounced ionization transition, as the effects of strong ionic microfields are effectively averaged out.

**Figure 5:**
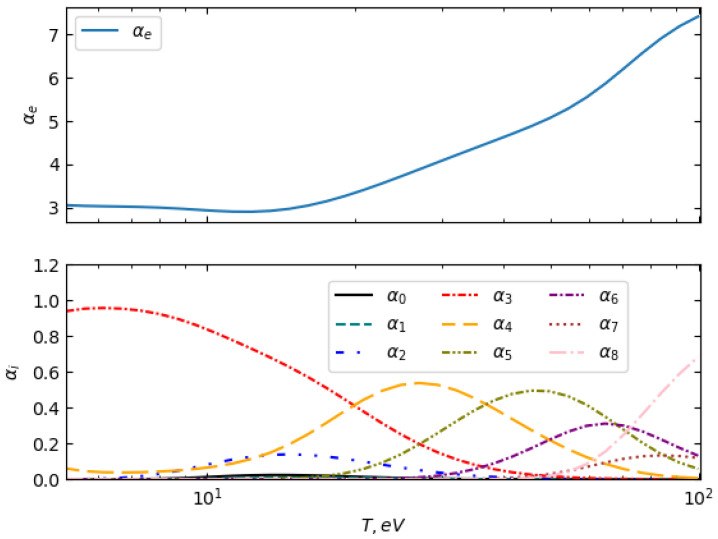
The composition of aluminum plasma at a solid density of $2.7\;g\;cm^{-3}$ as a function of plasma temperature, showing the evolution of ionization states with increasing temperature.

Figure 5 presents the ionization degree $\alpha_{e}$ (top panel) and the fractions of ionized species up to $\alpha_{8}$ (bottom panel) as functions of temperature in the range of 5 eV to 100 eV at the solid density of 2.7 $g\;cm^{-3}$. At lower temperatures (T≈5eV), the plasma is predominantly in the $\alpha_{3}$ ionization state, indicating a relatively high degree of ionization even at the lower boundary of the studied range. As temperature increases, higher charge states appear, with $\alpha_{4}$ and $\alpha_{5}$ becoming more significant around T≈10−20eV. Beyond T≈30eV, the plasma exhibits a continuous transition towards higher ionization states, with $\alpha_{6}$ and $\alpha_{7}$ fractions increasing, and the state $\alpha_{8}$ becoming dominant at T≳80eV. The overall behavior of the charge state distribution follows the expected trends observed in previous theoretical and experimental studies, confirming the validity of the applied model [8,44].

## 4. Conclusions

In this paper, we have presented an improved model for ionization potential depression in dense and strongly coupled plasmas, based on extending the dynamical structure factor (SF) formalism. Specifically, we replaced the Wigner–Seitz radius with an ion-sphere radius to better capture the local nature of individual ionization events, accounted for electron degeneracy through an interpolation between Debye–Hückel and Thomas–Fermi regimes, and implemented an iterative Saha equation solver that self-consistently incorporates these refinements. Comparisons with experimental data on warm dense aluminum demonstrate that these modifications yield more accurate IPD predictions than standard mean-field approaches such as Stewart–Pyatt, particularly in regimes where microfield fluctuations and partial degeneracy play dominant roles.

While the present model has successfully reproduced sharper ionization transitions in strongly coupled plasmas over a broad parameter space, some simplifying assumptions limit its scope. Here, we used ground-state partition functions and focused on single-element systems in local thermodynamic equilibrium (LTE). These choices can introduce uncertainties at elevated temperatures and in more complex mixtures, where excited ionic states and multi-species screening become relevant. Likewise, the assumption of LTE may not strictly hold for rapidly evolving laser-produced plasmas or other non-equilibrium conditions.

Furthermore, we note that the assumption of a spatially uniform electron background can become questionable in strongly coupled or inhomogeneous regimes. Recent studies (see, for example, [45]) have shown that free-electron partition functions may vary significantly with local conditions, shifting ionization equilibria toward lower charge states. In our work, using the ion-sphere radius rather than the Wigner–Seitz radius already provides a more localized description of the plasma environment. However, a fully non-uniform treatment of the free-electron distribution would require a more advanced, spatially resolved framework beyond the scope of this model. Such an extension could further refine predictions of IPD and charge-state distributions in dense, strongly coupled plasmas.

Future research will extend this framework in several directions. First, the model will be employed to calculate the electrical conductivity of dense plasmas, allowing us to connect the improved IPD estimates to dynamic transport properties that are crucial in high-energy-density physics. Second, we intend to incorporate non-ground-state partition functions, thus capturing the contributions of excited levels and further increasing the accuracy at higher temperatures. Finally, we will explore multi-component and non-LTE regimes, where the interplay of different ion species and time-dependent ionization processes demands a more generalized approach. Overall, the results presented here illustrate that a nuanced treatment of microfield fluctuations and electron degeneracy is essential for accurately describing the ionization and thermodynamic properties of warm and hot dense plasmas, which, in turn, supports the interpretation of experiments and simulations in fields ranging from astrophysics to inertial confinement fusion.

## Data Availability

The raw data supporting the conclusions of this article will be made available by the authors on request.
